# Self-Vibration of Liquid Crystal Elastomer Strings under Steady Illumination

**DOI:** 10.3390/polym15163483

**Published:** 2023-08-20

**Authors:** Haiyang Wu, Yuntong Dai, Kai Li

**Affiliations:** School of Civil Engineering, Anhui Jianzhu University, Hefei 230601, China; hywu@stu.ahjzu.edu.cn (H.W.); daiytmechanics@ahjzu.edu.cn (Y.D.)

**Keywords:** self-vibration, liquid crystal elastomer, light-driven, string

## Abstract

Self-vibrating systems based on active materials have been widely developed, but most of the existing self-oscillating systems are complex and difficult to control. To fulfill the requirements of different functions and applications, it is necessary to construct more self-vibrating systems that are easy to control, simple in material preparation and fast in response. This paper proposes a liquid crystal elastomer (LCE) string–mass structure capable of continuous vibration under steady illumination. Based on the linear elastic model and the dynamic LCE model, the dynamic governing equations of the LCE string–mass system are established. Through numerical calculation, two regimes of the LCE string–mass system, namely the static regime and the self-vibration regime, are obtained. In addition, the light intensity, contraction coefficient and elastic coefficient of the LCE can increase the amplitude and frequency of the self-vibration, while the damping coefficient suppresses the self-oscillation. The LCE string–-mass system proposed in this paper has the advantages of simple structure, easy control and customizable size, which has a wide application prospect in the fields of energy harvesting, autonomous robots, bionic instruments and medical equipment.

## 1. Introduction

Self-vibration exists widely in nature and engineering [1,2,3,4,5,6,7]. It is a non-attenuating vibration in which the process of vibration is accompanied by some periodically varying force by which the vibrating system can be replenished with energy to maintain the vibration. A self-vibration system usually includes vibration elements, steady energy sources and feedback mechanisms. Unlike forced vibration [8], self-vibration can independently obtain energy from the external steady environment to maintain its continuous vibration without additional periodic excitation. As a representative of nonlinear systems, self-vibration deepens the understanding of nonequilibrium dynamical processes [9,10], and also has guiding significance for constructing synchronous systems [11,12,13] and chaotic systems [14,15,16]. Self-vibration has autonomy, which is helpful to the design of autonomous components such as autonomous robots [17] and actuators [18,19]. Furthermore, self-vibration has significant application value in energy harvesting [20,21], soft robots [22,23], sensors [24], medical equipment [25,26] and other fields. 

In recent years, many efforts have been made to construct self-vibration systems, among which a self-vibration system based on active materials has attracted extensive research interest. Active materials are kinds of material that can change their shapes or motion states when they are stimulated by external stimuli such as light [27,28], heat [29,30], electricity [31], magnetism [32,33] and so on. Common active materials include hydrogels [34], ionic gels [35,36], photoresponsive or thermal responsive polymers [37,38,39,40,41,42,43], dielectric elastomers [44], shape memory polymers [45] etc. Based on the response of active materials to external steady stimuli, people have built a variety of self-vibration modes, such as bending [46,47], swinging [48], rolling [49,50], twisting [51], vibrating [52], floating [53], buckling [54,55], jumping [56], stretching [57], shuttling [58], spinning [59] and curling [60]. In addition, some ingenious feedback mechanisms have been carefully designed, such as the self-shading mechanism [61], coupling mechanism of large deformation and chemical reaction [36], coupling mechanism of liquid volatilization and deformation [62] and photothermal surface tension gradient [63,64] to break the balance of the system, which leads to a stable and sustained response of the active material and further generates self-vibration. 

Among these active materials that can be used to construct self-vibration systems, liquid crystal elastomers (LCEs) are widely considered because of their unique advantages. LCE is a unique material that is a liquid crystal polymer with a network structure formed after moderate cross-linking mesogens [65]. Its unique properties combine the characteristics of liquid crystals and elastomers, and it is capable of demonstrating an amazing ability to change shape. LCE presents a host of advantages, including substantial and reversible deformability [66,67], rapid deformation and straightforward controllability, which have garnered significant attention among researchers. Due to the rotation or phase transitions of liquid crystal monomer, liquid crystal elastomers can show reversible morphological changes when subjected to external stimuli, such as light [27,28], heat [29,30], electricity [31], magnetism [32,33] etc., displaying a variety of shapes and structures. Among these external stimuli, light stands out due to its fast response, environmental friendliness, easy accessibility, noiselessness [15,68,69] and precise control [70]. Considering the advantages of light, a rich variety of self-vibration systems based on light-driven LCE have been developed, such as bending [46,47], buckling [54,55], jumping [56], swimming [48] and other self-vibration systems. These self-vibration systems based on light-driven LCE have broad application prospects in bionic instruments [71], energy harvesting [20,21], actuators [19], soft robots [22,23] and other fields.

Although self-vibrating systems based on LCE have been widely developed, the design and construction of LCE self-vibration systems still have great limitations, such as complex structure, difficulty to control and difficulty to prepare. Therefore, it is necessary to construct more LCE self-vibration structures with simple structures and that are controllable and convenient. The tension string system has been widely studied as a classical self-vibration system. In this paper, we creatively propose a new self-vibrating system that is different from the previous self-vibration systems, which consists of two LCE strings and a mass block, and which can obtain sustained and stable vibration under steady illumination. Compared with existing self-oscillating systems [52,53], the system proposed by us has a simpler structure and is easier to implement. Our goal is to construct novel self-oscillating systems based on active materials that are simple in structure, easy to control, customizable in size and easy to prepare. Also, the effect of system parameters on self-oscillation is discussed to provide guidance for regulating this system. Depending on its excellent properties, the self-oscillating LCE string–mass system has significant application value in autonomous actuators, energy collectors, bionic instruments and other fields. 

The paper is as follows. Firstly, in Section 2, based on the LCE dynamic model, a theoretical model of the LCE string system is established and the corresponding governing equations are derived. Then, in Section 3, two motion regimes of the LCE string system are described and the mechanism of self-vibration is explained in detail. In Section 4, the effects of system parameters on the amplitude and frequency of self-vibration are further discussed quantitatively. Finally, in Section 5, the results of this paper are summarized.

## 2. Theoretical Model and Formulation

In this section, firstly, a novelty light-driven self-vibration system consisting of two LCE strings and a mass block is described. Secondly, we derive the governing equations of the self-vibration system based on the dynamic LCE model, vibration theory and Newton’s second law. Finally, the governing equation is dimensionless and the numerical calculation method is introduced. 

### 2.1. Dynamics of Self-Vibration of LCE Strings

**Figure 1:**
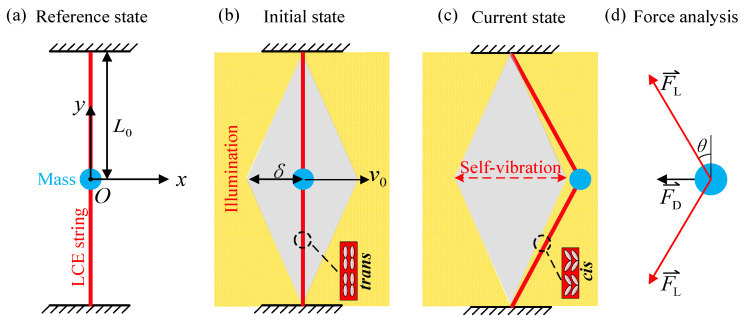
Diagram of self-vibration system of LCE strings: (**a**) Reference state; (**b**) Initial state; (**c**) Current state; (**d**) Force analysis. The mass block is subjected to the tension $F_{L}$ of the LCE strings and the air damping force $F_{D}$. The LCE string–mass system can vibrate continuously and periodically under steady illumination.

Figure 1 shows the physical model of the system of light-driven LCE self-vibration. The system, which can vibrate continuously and steadily under a given initial speed and designed illumination condition, consists of two LCE strings and a mass block, as shown in Figure 1a. One end of each LCE string is fixed to a horizontal rigid base, and the other end is attached to the mass block with a mass of m. The original length of each LCE string in the unstressed state is $L_{0}$. Considering that the gravity mg on the mass block is much less than the elastic force on it, the gravity is ignored. We take the initial position of the mass block as the origin of the coordinate system, with the horizontal direction as the x-axis and the vertical direction as the y-axis, as shown in Figure 1a. Since the two LCE strings are exactly the same, the tension of the two LCE strings is exactly the same, and the tension generated by the two strings in the vertical direction cancels each other, so the mass block only vibrates in the horizontal direction, and its displacement is x. 

As shown in Figure 1b,c, the yellow area represents the illumination zone and the gray rhomboid area represents the shading (non-illumination zone); the distance from the right end of the shading zone to the origin is δ. Due to the action of the initial velocity, the mass block continues to move to the right until it reaches the illuminated zone. UV light radiation can change the photochromic liquid crystal molecules in the material from straight trans configuration to bent cis configuration [65]. Thus, under continuous illumination, the chromophores (azobenzene) in the LCE fibers absorb light energy, followed by a continuous cycle of cis–trans isomerization. This process results in the transformation of the chromophores from the trans state to the cis state, thereby inducing the contraction of the LCE fibers. With the contraction and stretching of the LCE strings in the illuminated zone, the elastic potential energy of the system reaches its peak when the mass block reaches the maximum distance. The next moment, under the action of the tension of the LCE strings, the mass block moves in the opposite direction. When moving into the non-illumination zone, the light-driven contraction of the LCE strings resumes, at which point the tension of the LCE strings decreases until it reaches the illuminated zone on the other side, accumulating elastic potential energy, and then repeating the process. The LCE string–mass system can maintain continuous and stable self-vibration through the choice of proper system parameters and initial conditions. 

The mass block is subjected to the tension of the LCE strings and the air damping force, as shown in Figure 1d. In the horizontal direction, the governing equation of mass block can be described as:

(1) $$m\ddot{x}(t)=-2F_{L}\mathrm{sgn}(x)\sin\theta -F_{D},$$

where $\ddot{x}$ represents the acceleration of the mass block, $F_{L}$ indicates the tension of the LCE strings, θ is the angle between the LCE strings and the y-axis, the sign function sgn(*x*) is a function that returns the sign of a real number *x*, and $F_{D}$ denotes the air damping force.

It can be obtained by geometric relations that $\sin\theta =\frac{x}{\sqrt{L_{0}^{2}+x^{2}}}$. where $L_{0}$ is the original length of each LCE string in the unstressed state and x indicates the horizontal displacement of mass block. 

The tension of the LCE strings is proportional to its elongation, with the formula being:

(2) $$F_{L}=K(\sqrt{L_{0}^{2}+x{\left(t\right)}^{2}}-L_{0}-L_{0}\varepsilon_{I}(t)),$$

where K denotes the elastic coefficient of the LCE and $\varepsilon_{I}$ refers to the light-driven contraction strain of the LCE strings. 

The damping force is assumed to be linearly proportional to the velocity of the mass block and can be expressed as:

(3) $$F_{D}=\beta \dot{x}(t),$$

where β represents the air damping coefficient and $\dot{x}$ denotes the velocity of the mass block.

Substituting Equations (2) and (3) into Equation (1), we can obtain:

(4) $$m\ddot{x}(t)=-2K(\sqrt{L_{0}^{2}+x{\left(t\right)}^{2}}-L_{0}-L_{0}\varepsilon_{I}(t))\cdot \sin\theta -\beta \dot{x}(t).$$

### 2.2. Dynamic LCE Model

This section mainly deduces the strain equation of the LCE strings under illumination and non-illumination conditions. A linear model is adopted to describe the relationship between the cis number fraction ϕ(t) in LCE and the light-driven contraction of the LCE, namely:

(5) $$\varepsilon_{I}=-C\phi (t),$$

where C indicates the contraction coefficient of the LCE. 

The light-driven contraction strain of the LCE strings depends on the cis number fraction ϕ(t) in the LCE. UV light radiation can change the photochromic liquid crystal molecules (azobenzene) in the material from straight trans configuration to bent cis configuration, which is often accompanied with the contraction of a monodomain LCE along the mesogen aligning direction [65]. For simplicity, the LC cis–trans switching is assumed to be strain-independent. Furthermore, according to Nagele et al. [72], the cis number fraction depends on the thermal excitation from trans to cis, the thermal drive relaxation from cis to trans and the light-driven trans to cis isomerization. Assuming that the thermal excitation from trans to cis can be ignored, the governing equation for the evolution of the number fraction can be expressed as:

(6) $$\frac{\partial \phi }{\partial t}=\eta_{0}I(1-\phi )-\frac{\phi }{T_{0}},$$

where $\eta_{0}$ represents the light absorption constant, $T_{0}$ represents the thermally driven relaxation time from the cis to trans and I indicates the light intensity. 

By solving Equation (6), we can get:

(7) $$\phi (t)=\frac{\eta_{0}T_{0}I}{\eta_{0}T_{0}I+1}+(\phi_{0}-\frac{\eta_{0}T_{0}I}{\eta_{0}T_{0}I+1})\exp\left[-\frac{t}{T_{0}}(\eta_{0}T_{0}I+1)\right],$$

where $\phi_{0}$ denotes the initial cis number fraction in non-illumination zone. 

In the illumination zone, the initial number fraction $\phi_{0}=0$, so Equation (7) can be simplified as:

(8) $$\phi (t)=\frac{\eta_{0}T_{0}I}{\eta_{0}T_{0}I+1}\left\{1-\exp\left[-\frac{t}{T_{0}}\left(1+\eta_{0}T_{0}I\right)\right]\right\}.$$

In the non-illumination zone, by setting the light intensity I=0, we can obtain:

(9) $$\phi \left(t\right)=\phi_{0}\exp\left(-\frac{t}{T_{0}}\right).$$

In this case, $\phi_{0}$ can be chosen as the maximum value of $\phi_{0}$ in Equation (8) under continuous illumination. Then we can obtain:

(10) $$\phi \left(t\right)=\frac{\eta_{0}T_{0}I}{\eta_{0}T_{0}I+1}\exp\left(-\frac{t}{T_{0}}\right).$$

### 2.3. Nondimensionalization

For ease of calculation, we define the following dimensionless quantities: $\bar{x}=x/L_{0}$, $\bar{\dot{x}}=\dot{x}T_{0}/L_{0}$, $\bar{\ddot{x}}=\ddot{x}T_{0}^{2}/L_{0}$, $\bar{t}=t/T_{0}$, $\bar{\delta }=\delta /L_{0}$, $\bar{I}=IT_{0}\eta_{0}$, $\bar{\beta }=\beta T_{0}/m$ and $\bar{K}=KT_{0}^{2}/m$. So, the dimensionless form of governing equation can be written as:

(11) $$\bar{\ddot{x}}(t)=-2\bar{K}(\sqrt{1+\bar{x}{\left(t\right)}^{2}}-1-\varepsilon_{I}(t))\frac{x}{\sqrt{1+\bar{x}{\left(t\right)}^{2}}}-\bar{\beta }\bar{\dot{x}}(t).$$

In the illuminated state, Equation (8) can be rewritten as:

(12) $$\bar{\phi }(t)=1-\exp\left[-\bar{t}(\bar{I}+1)\right].$$

In the non-illumination zone, we can obtain:

(13) $$\bar{\phi }(t)=\exp(-\bar{t}).$$

Obviously, Equation (11) is a second-order nonlinear differential equation, and it is difficult to find the analytical solution of this kind of equation. Therefore, we use Matlab software (version R2018b) and the four-order Runge–Kutta method for numerical calculation. By adjusting the parameters within the program, for example $\bar{I}$, C, $\bar{K}$, ${\bar{v}}_{0}$, $\bar{\beta }$ and $\bar{\delta }$, we can obtain the displacement, velocity, elastic force, damping force and light-driven contraction strain of the self-vibration of the LCE string–mass system at each moment. 

## 3. Two Motion Regimes and Mechanism of Self-Vibration

In this section, firstly, two typical motion regimes of the LCE string–mass system are described, namely the static regime and the self-vibration regime. Secondly, the corresponding mechanism of the self-vibration is elaborated in detail. 

### 3.1. Two Motion Regimes

To study the self-vibration of the LCE string–mass system, it is necessary to calculate the typical values of the dimensionless system parameters. According to the existing experimental [72,73,74,75] and research results, the actual values of each system parameter are summarized in Table 1, and the corresponding dimensionless system parameters are listed in Table 2. 

**Figure 2:**
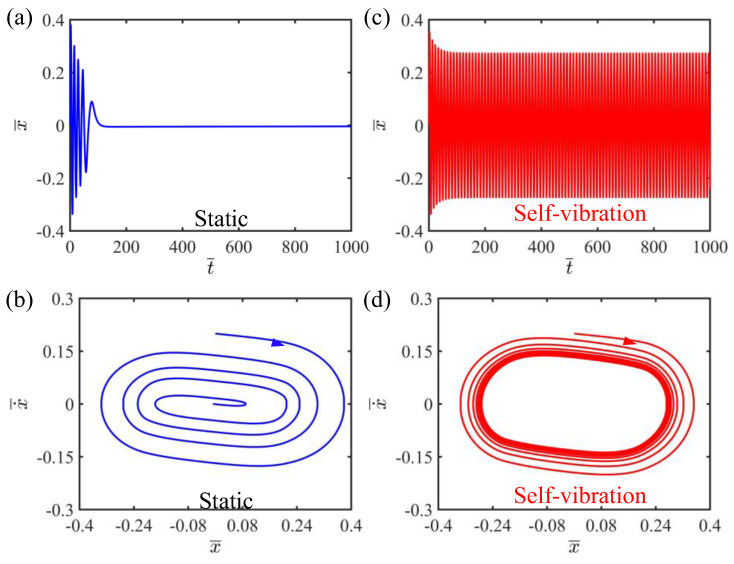
Two typical regimes: static regime and self-vibration regime. (**a**) Time history curve of the displacement with $\bar{I}=0.2$; (**b**) Phase trajectory diagram with $\bar{I}=0.2$; (**c**) Time history curve of the displacement with $\bar{I}=0.5$; (**d**) The limit cycle in phase diagram with $\bar{I}=0.5$. Two kinds of system regimes can be obtained with different light intensities, namely static regime and self-vibration regime.

Through the numerical solution of Equation (11), the time history curve vibration and phase trajectory diagram of the LCE string–mass system can be obtained, as shown in Figure 2. In this case, the other system parameters in the numerical calculation are set as C=0.25, $\bar{K}=2$, ${\bar{v}}_{0}=0.2$, $\bar{\beta }=0.1$ and $\bar{\delta }=0.2$. As can be seen from Figure 2, the system of the LCE strings has two different regimes, namely the static regime and the self-vibration regime. Figure 2a,b depict the static regime, where the vibration of the system finally stops and the corresponding phase trajectory diagram terminates at a point. In contrast, Figure 2c,d plot the self-vibration regime, in which the vibration of the system tends to stabilize after a period of time and maintains a fixed amplitude and period, and a limit cycle representing a single periodic motion appears in the corresponding phase trajectory diagram. The reason for the self-vibration phenomenon is that the system obtains enough light energy to compensate for the damping dissipation, so as to maintain its self-sustained vibration. The emergence of the phenomenon of self-vibration proves the rationality and feasibility of our constructed system. In the next section, we will elaborate on the mechanism of the self-vibration phenomenon.

### 3.2. Mechanism of Self-Vibration

**Figure 3:**
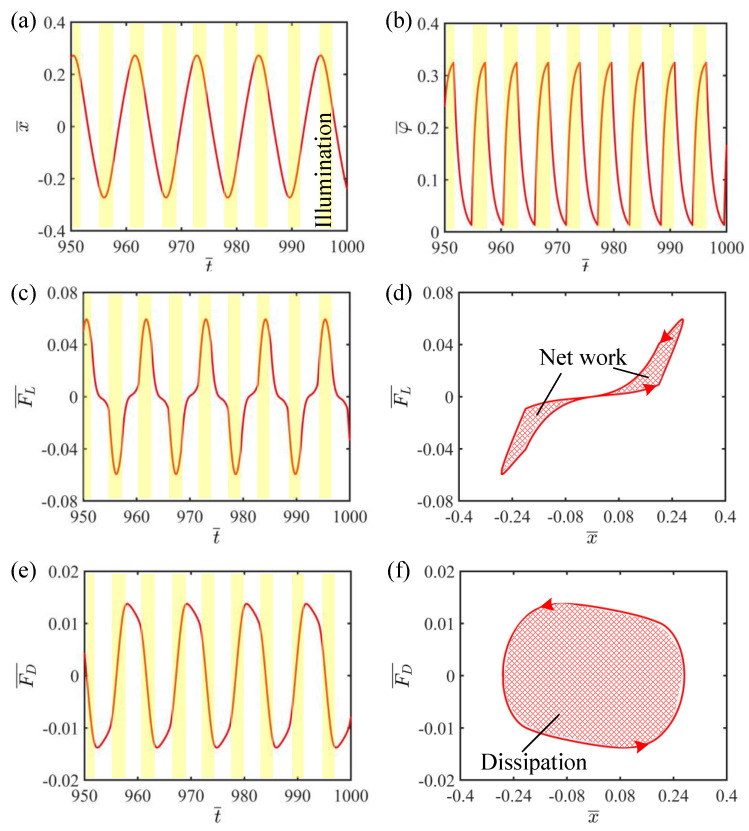
The mechanism of self-vibration of the LCE string–mass system. (**a**) Time history curve of the displacement. (**b**) Time history curve of the number fraction. (**c**) Variation of the tension of LCE strings with time. (**d**) Dependence of the tension of LCE strings on the displacement. (**e**) Variation of the damping force with time. (**f**) Dependence of the damping force on the displacement. The work done by the elastic force can compensate the damping dissipation, so the system can maintain stable vibration.

**Figure 4:**
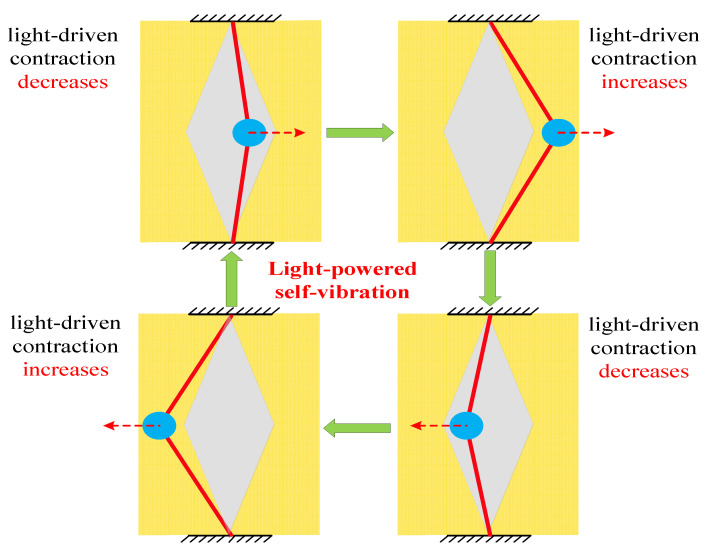
Snapshots of the LCE string–mass system in one cycle during the self-vibration. Under steady illumination, the system exhibits a continuous periodic self-vibration.

This section aims to explain the mechanism of self-vibration, that is, the energy compensation mechanism of the LCE string–mass system. To better understand the energy compensation mechanism, it is necessary to plot the change curves of some key physical quantities in the process of self-vibration, as shown in Figure 3. In this case, the dimensionless parameters of the system are selected as $\bar{I}=0.5$, C=0.25, $\bar{K}=2$, ${\bar{v}}_{0}=0.2$, $\bar{\beta }=0.1$ and $\bar{\delta }=0.2$. Figure 3a shows the curve of the mass block horizontal displacement over time, where the yellow area indicates the LCE strings are illuminated. It can be easily found that the LCE string–mass system at this time maintains a stable amplitude and period, and the mass block shuttles in the illumination zone on both sides. Figure 3b plots that when the displacement of the mass block is greater than the width of shade $\bar{\delta }$, the LCE strings are in the illumination zone, and the number fraction in the LCE gradually increases and tends to a limit value. When the displacement of the mass block is less than the width of shade $\bar{\delta }$, the LCE strings are in the non-illumination zone, and the number fraction in the LCE rapidly decreases to zero. As the mass block regularly enters and exits the illumination zone, the number fraction in the LCE strings also changes periodically. In addition, Figure 4 illustrates several characteristic snapshots for the self-vibration of the LCE string–mass system during one cycle under steady illumination. 

In order to understand the energy source and consumption of the LCE string–mass system, we plot the elastic force and damping force of the LCE with time and with displacement, as shown in Figure 3c–f. Figure 3c shows the variation of tension of the LCE strings over time. With the periodic vibration of the LCE string–mass system, the variation of tension of the LCE strings is also periodic. When the LCE strings enter the illumination zone, the tension of the LCE strings increases due to the light-driven contraction of the LCE strings. When the LCE strings leave the illumination zone, the light-driven contraction of the LCE strings recovers and the tension of the LCE strings decreases, as shown in Figure 3c. Figure 3d shows the hysteresis loop of the tension of the LCE string, the area of which represents the net work done by the tension of the LCE strings in one cycle of vibration, which is numerically calculated to be 0.018. Similarly to the tension of the LCE strings, Figure 3e plots the periodic change of damping force with time. Figure 3e shows the relationship between the damping force and displacement, and the hysteresis loop enclosed represents the work done by the damping force in one cycle of vibration, that is, the system damping dissipation. Through calculation, the area of the hysteresis loop in Figure 3f is also 0.018, which means that the energy lost by air damping during the self-vibration is compensated for by the work done by the tension of the LCE strings. Therefore, the self-vibration of the LCE string–mass system can be sustained. 

## 4. Parametric Study

In this section, we quantitatively investigate the effects of system parameters such as light intensity, contraction coefficient, elastic coefficient, initial velocity, damping coefficient and width of shade on the amplitude *A* and frequency *F* of the self-vibration of the LCE string–mass system. 

### 4.1. Effect of the Light Intensity

**Figure 5:**
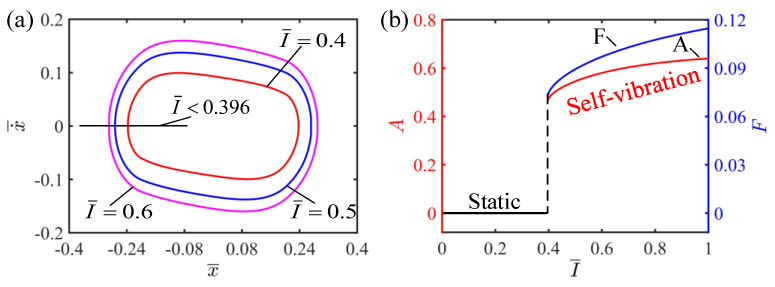
The effect of light intensity on the self-vibration. (**a**) Limit cycles with $\bar{I}=0.4$, $\bar{I}=0.5$ and $\bar{I}=0.6$. (**b**) Variations of amplitude and frequency with different light intensities. The larger the light intensity, the larger the amplitude and frequency of self-vibration.

The light intensity influencing the self-vibration of the LCE string–mass system is investigated in this section. In this case, the values of the other parameters are C=0.25, $\bar{K}=2$, ${\bar{v}}_{0}=0.2$, $\bar{\beta }=0.1$ and $\bar{\delta }=0.2$. Figure 5a plots the limit cycles of self-vibration for $\bar{I}=0.4$, $\bar{I}=0.5$ and $\bar{I}=0.6$. The horizontal width of the limit cycle represents the amplitude of the self-vibration, and the vertical height of the limit cycle indicates the velocity of the self-vibration. It can be seen from Figure 5a that the limit cycle is the largest for $\bar{I}=0.6$, which indicates that the amplitude and kinetic energy of the self-vibration are largest in this case. Figure 5b shows the effect of the light intensity on amplitude and frequency. When the light intensity is below 0.396, the LCE strings cannot absorb enough light energy to offset the damping dissipation, and therefore cannot maintain continuous motion, thus entering a static state. When the light intensity is higher than 0.396, the LCE strings are able to absorb enough light energy to offset the damping dissipation and thus maintain a continuous stable vibration, i.e., the self-vibration regime. In the regime of self-vibration, the amplitude and frequency increase with the increase in light intensity. This is because the higher the light intensity, the greater the contraction and the greater the tension of the string. The greater tension is able to do more work on the system, producing more kinetic energy and thus a greater amplitude. The above results show that increasing the light intensity can make the light-driven system absorb more energy to achieve a larger amplitude, which is consistent with the current findings [3]. 

### 4.2. Effect of the Contraction Coefficient of LCE

**Figure 6:**
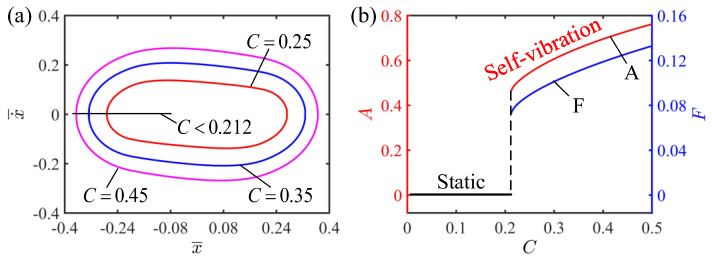
The effect of contraction coefficient on the self-vibration. (**a**) Limit cycles with C=0.25, C=0.35 and C=0.45. (**b**) Variations of amplitude and frequency with different contraction coefficients. The larger contraction coefficient, the larger the amplitude and frequency of self-vibration.

This section presents a discussion on the effect of the contraction coefficient on the self-vibration of the LCE strings. Here, the values of the other parameters are $\bar{I}=0.5$, $\bar{K}=2$, ${\bar{v}}_{0}=0.2$, $\bar{\beta }=0.1$ and $\bar{\delta }=0.2$. Figure 6a plots the limit cycles of the self-vibration of the LCE string–mass system with different contraction coefficients. It can be observed from Figure 6a that the limit cycle with a larger contraction coefficient completely wraps the limit cycle with a smaller contraction coefficient, indicating that the larger the contraction coefficient, the larger the energy of the LCE string–mass system, and thus the larger the amplitude and kinetic energy. It can be seen from Figure 6b that the amplitude and frequency of the self-vibration change with the change of the contraction coefficient. When the contraction coefficient is less than 0.212, the system is in the static regime. On the contrary, when the contraction coefficient is greater than 0.212, the system is in the self-vibration regime. With the increase in the contraction coefficient, the amplitude and frequency also increase. The reason for this phenomenon is similar to the reason for the effect of light intensity on self-vibration: when the contraction coefficient is small, the LCE strings absorb insufficient light energy when they are in the illumination zone, and cannot obtain enough energy to compensate for the damping dissipation, so that the system eventually moves to the static regime. When the contraction coefficient is large, the LCE strings absorb enough energy in the illumination zone and have enough energy to compensate for the damping dissipation of the system, so as to maintain the self-vibration. As the contraction coefficient continues to increase, the light energy absorbed by the LCE strings further increases, and so does the amplitude.

### 4.3. Effect of the Elastic Coefficient of LCE

**Figure 7:**
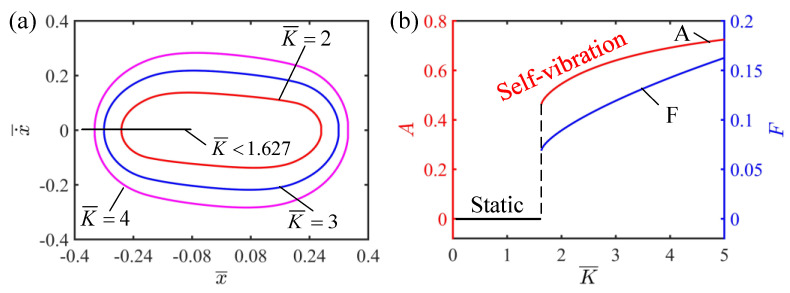
The effect of elastic coefficient on the self-vibration. (**a**) Limit cycles with $\bar{K}=2$, $\bar{K}=3$ and $\bar{K}=4$ (**b**) Variations of amplitude and frequency with different elastic coefficients. The larger the elastic coefficient, the larger the amplitude and frequency of self-vibration.

This section provides the influence of the elastic coefficient of the LCE strings on the self-vibration for $\bar{I}=0.5$, C=0.25, ${\bar{v}}_{0}=0.2$, $\bar{\beta }=0.1$ and $\bar{\delta }=0.2$. Figure 7a shows the limit cycle for different elastic coefficients of the LCE strings. When the elastic coefficient is less than 1.627, the phase trajectory diagram of the self-vibration is a fixed point, which indicates that the system is in the static regime. This is because when the elastic coefficient is small, the tension generated by the LCE strings in the illumination zone is small, which cannot provide enough elastic potential energy to compensate for the damping dissipation of the system, so the system finally reaches a static state. It can be seen from Figure 7b that the elastic coefficient has a significant influence on the amplitude and frequency of the self-vibration. With the increase in the elastic coefficient, the amplitude and frequency of the self-vibration increase. This is because as the elastic coefficient increases, the elastic force generated by the LCE strings increases, the elastic potential energy that the system is able to convert into kinetic energy increases, and therefore the amplitude of the self-vibration increases. Therefore, in the design of a tension system based on LCEs, it is key to select the appropriate elastic coefficient to obtain better performance. 

### 4.4. Effect of the Initial Velocity

**Figure 8:**
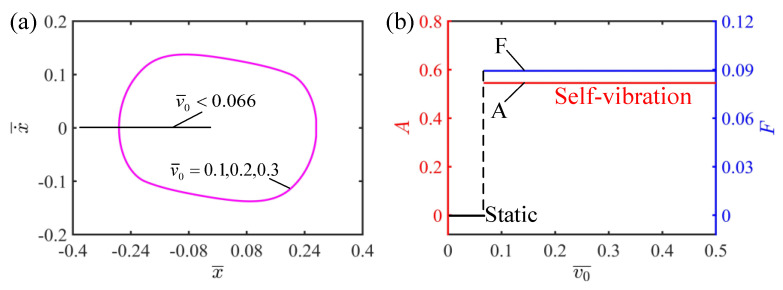
The effect of initial velocity on the self-vibration. (**a**) Limit cycles with ${\bar{v}}_{0}=0.1$, ${\bar{v}}_{0}=0.2$ and ${\bar{v}}_{0}=0.3$. (**b**) Variations of amplitude and frequency with different initial velocities. The initial velocity has no effect on the amplitude and frequency of self-vibration.

This section mainly focuses on the effect of initial velocity on the self-vibration of the LCE string–mass system, with parameters $\bar{I}=0.5$, C=0.25, $\bar{K}=2$, $\bar{\beta }=0.1$ and $\bar{\delta }=0.2$. It can be observed from Figure 8a that the self-vibration can be successfully triggered at ${\bar{v}}_{0}=0.1$, ${\bar{v}}_{0}=0.2$ and ${\bar{v}}_{0}=0.3$. It is worth mentioning that the limit cycles at different velocities coincide completely. Figure 8b plots the relationship between the initial velocity and the amplitude and frequency of the self-vibration. It can be seen from Figure 8b that when the initial velocity is less than 0.066, the system is in the static regime, because the low initial velocity cannot allow the LCE strings to reach the illumination zone to absorb enough light energy, and it finally reaches the static regime. When the initial velocity is greater than 0.066, the system is in the self-vibration regime and the final amplitude and frequency are not affected. This is because the amplitude of the self-vibration depends on the energy conversion between the work done by the LCE strings and the damping dissipation, which belongs to the internal characteristics of the system, and the initial velocity does not affect the energy conversion of the system, so the amplitude does not change. Compared with other parameters, the initial velocity is more like a switch that triggers the self-vibration, which is responsible only for activating the system and does not affect the inherent characteristics of the system such as amplitude and frequency, which is in agreement with the results of existing studies [59]. 

### 4.5. Effect of the Damping Coefficient

**Figure 9:**
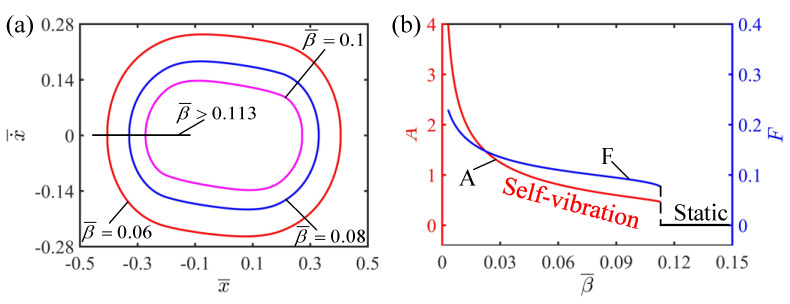
The effect of the damping coefficient on the self-vibration. (**a**) Limit cycles with $\bar{\beta }=0.06$, $\bar{\beta }=0.08$ and $\bar{\beta }=0.1$. (**b**) Variations of amplitude and frequency with different damping coefficients. The larger the damping coefficient, the larger the amplitude and frequency of self-vibration.

This section mainly studies the damping coefficient on the self-vibration of the LCE string–mass system. In the calculation, we set $\bar{I}=0.5$, C=0.25, $\bar{K}=2$, ${\bar{v}}_{0}=0.2$ and $\bar{\delta }=0.2$. The damping coefficient has a significant effect on the regime and amplitude of the system, as shown in Figure 9. Figure 9a draws the limit cycles for $\bar{\beta }=0.06$, $\bar{\beta }=0.08$ and $\bar{\beta }=0.1$. It can be seen from Figure 9a that the smaller the damping coefficient is, the larger the limit cycle is. It can be seen from Figure 9b that there is a critical value between the static regime and the self-vibration regime. The system is in the static regime for $\bar{\beta }>0.113$, while the system is in the self-vibration regime for $\bar{\beta }<0.113$. In addition, it can be seen from Figure 9b that the smaller the damping coefficient is, the larger the amplitude and frequency of the self-vibration are. This can be explained in terms of energy compensation. The greater the damping coefficient, the greater the damping force of the system, which hinders the movement of the system and makes the LCE strings unable to reach the illuminated zone to absorb light energy. The energy of the system decreases continuously due to damping dissipation, so the system eventually reaches a static state. On the contrary, the smaller the damping coefficient, the smaller the damping dissipation of the system and the larger the converted kinetic energy, and thus the amplitude increases. Therefore, how to reduce the damping dissipation of the system through reasonable structural design is an important challenge. 

### 4.6. Effect of the Width of Shade

**Figure 10:**
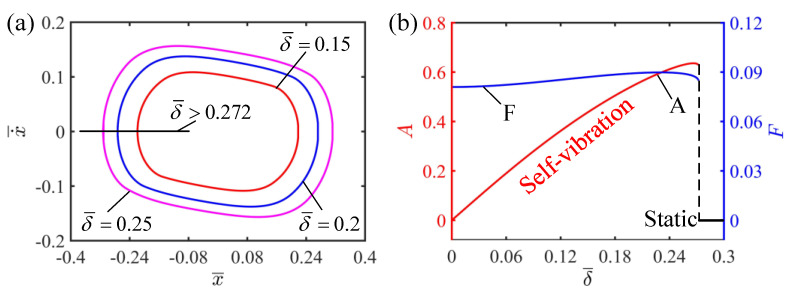
The effect of the width of shade on the self-vibration. (**a**) Limit cycles with $\bar{\delta }=0.15$, $\bar{\delta }=0.2$ and $\bar{\delta }=0.25$. (**b**) Variations of amplitude and frequency with different widths of shade. The larger the width of shade, the larger the amplitude of self-vibration.

The effect of the width of shade on the self-vibration is discussed in the current section. In this case, the other dimensionless parameters are selected as $\bar{I}=0.5$, C=0.25, $\bar{K}=2$, ${\bar{v}}_{0}=0.2$ and $\bar{\beta }=0.1$. It is not difficult to find that the width of shade affects the motion regime of the system. Figure 10a plots the limit cycle with different widths of shade. It can be seen from Figure 10b that there is a critical value between the static regime and the self-vibration regime. When the width of shade is greater than 0.272, the system cannot reach the illumination zone to absorb light energy, the initial kinetic energy is constantly consumed and finally the system reaches the static state. When the shadow width is less than 0.272, the system can reach the illumination to absorb light energy to compensate for the damping dissipation, so it can continue stable vibration, namely, the self-vibration regime. Figure 10b shows that, in the self-vibration regime, the amplitude of the system increases with the increase in the width of shade. This is because when the width of shade is small, the LCE strings soon enter the illumination zone, the elastic force of the LCE strings rapidly increases and inhibits the further displacement of the mass block, and thus the amplitude of the self-vibration is small. On the other hand, when the width of shade is large, there is larger displacement before the LCE strings enter the illumination zone, so that the whole amplitude of the self-vibration is larger. 

## 5. Conclusions

The self-vibration system can directly absorb energy from the steady external environment to maintain its continuous motion without external periodic stimuli, which has great application prospects in the fields of autonomous robotics, energy harvesting and bionic devices. Traditional self-vibration systems have the defects of complex structure, difficult manufacturing and poor controllability, so there is a great necessity to construct new self-vibration systems. In this paper, we construct a new self-vibration system, which consists of two LCE strings and a mass block, and it can achieve continuous and stable vibration under steady illumination. Based on the dynamic LCE model and linear elastic model, the theoretical model of self-vibration of the LCE string–mass system is established and the corresponding governing equations are derived. Based on the results of numerical simulations, two motion regimes of the LCE string–mass system, namely the static regime and self-vibration regime, are described, and the energy compensation mechanism of the self-vibration is revealed. In addition, the effects of the system parameters on the amplitude and frequency of the self-vibration are quantitatively discussed. The results show that the amplitude and frequency of the system increase with the increase in light intensity, contraction coefficient and elastic coefficient. By adjusting these coefficients, it is expected that faster, more powerful active machines can be realized. The damping coefficient inhibits the amplitude and frequency of the self-vibration, while the initial velocity does not affect the amplitude and frequency of the self-vibration regimes. Meanwhile, the values of the parameters can also determine the motion modes of the system, and there are critical values between the self-vibrating and static modes. In addition, future goals are to increase the credibility of our findings through experiments, as well as to build more active machines based on active materials and to realize their applications in fields such as energy harvesting, artificial muscles and autonomous robotics. The research in this paper deepens the understanding of self-vibration systems and helps to design new self-vibration systems. Meanwhile, the LCE string–mass system proposed in this paper has the advantages of simple structure, easy control and customizable size, and has the prospect of application in the field of autonomous robots and bionic instruments. 

## Figures and Tables

**Table 1:** Material properties and geometric parameters.

Parameter	Definition	Value	Unit
I	light intensity	0~100	kW/m^2^
C	contraction coefficient	0~0.5	/
K	elastic coefficient	1~50	N/m
$T_{0}$	thermally driven relaxation time	0.01~0.5	s
$\eta_{0}$	light absorption constant	0.00022	m^2^/s·W
m	mass	0~2	kg
β	damping coefficient	0~0.5	kg/s
$v_{0}$	initial velocity	0~0.4	m/s
δ	width of shade	0~0.1	m
$L_{0}$	original length of LCE	0.01~0.2	m

**Table 2:** Dimensionless parameters.

**Parameter**	$\bar{I}$	C	$\bar{K}$	${\bar{v}}_{0}$	$\bar{\beta }$	$\bar{\delta }$
**Value**	0~1	0~0.5	0~10	0~1	0~0.2	0~0.5

## Data Availability

The data that support the findings of this study are available upon reasonable request from the authors.
